# Supplementary material for: DNA‐Barcoded Polysaccharide Specific Monoclonal Antibodies Facilitate Sensitive and Multiplexed Detection of Cell Wall Polymers
Source: Plant Biotechnol J. 2026 Oct 28:10.1111/pbi.70779. Online ahead of print. doi: 10.1111/pbi.70779 (PMC13715374; doi:10.1111/pbi.70779)
Supplement: Supplementary file 1 [file PBI-9999-0-s001.pdf]

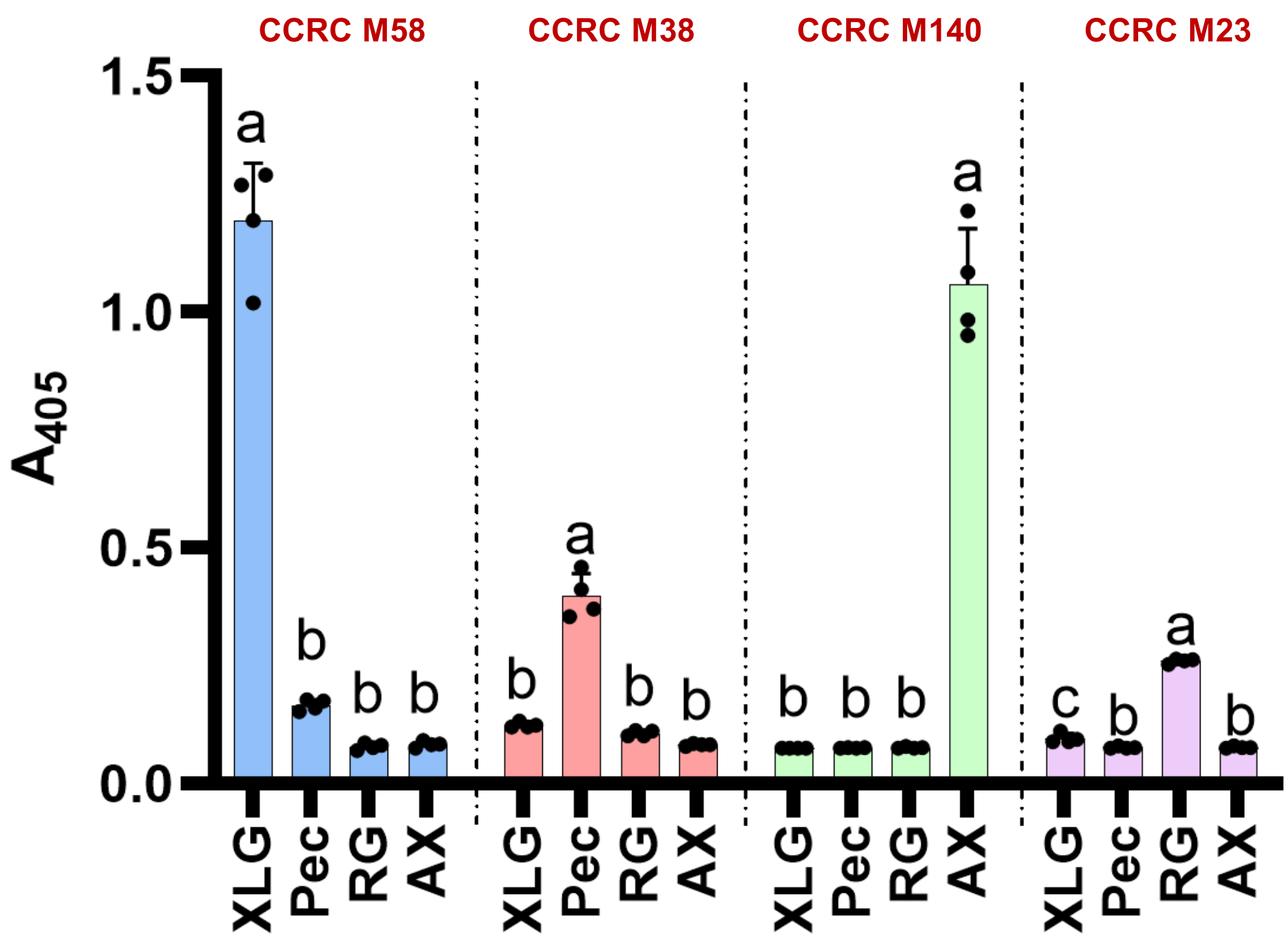

Supplemental Figure S1: ELISA-based cross-reactivity assays of CCRC mAbs considered in this study: 96 well plates were coated with 100 ng of Tamarind Xyloglucan (XLG), Apple pectin (Pec), Soybean RG-I (RG), or Wheat Arabinoxylan (AX). Wells were blocked and probed with 1:10 dilutions of CCRC-M58 (blue bars), CCRC-M38 (red bars), CCRC-M140 (green bars), or CCRC-M23 (magenta bars) hybridoma supernatant. ELISA signals were generated by a goat anti-mouse alkaline phosphatase conjugated secondary antibody as described in Materials and Methods. Data are plotted with all data points shown. Error bars represent SD ($n = 4$). Letters above bars represent statistically different categories as determined by One-Way ANOVA analysis of individual antibody groups followed by Tukey's post-hoc analysis ($P<0.001$). Samples sharing the same letter are not statistically different from one another.

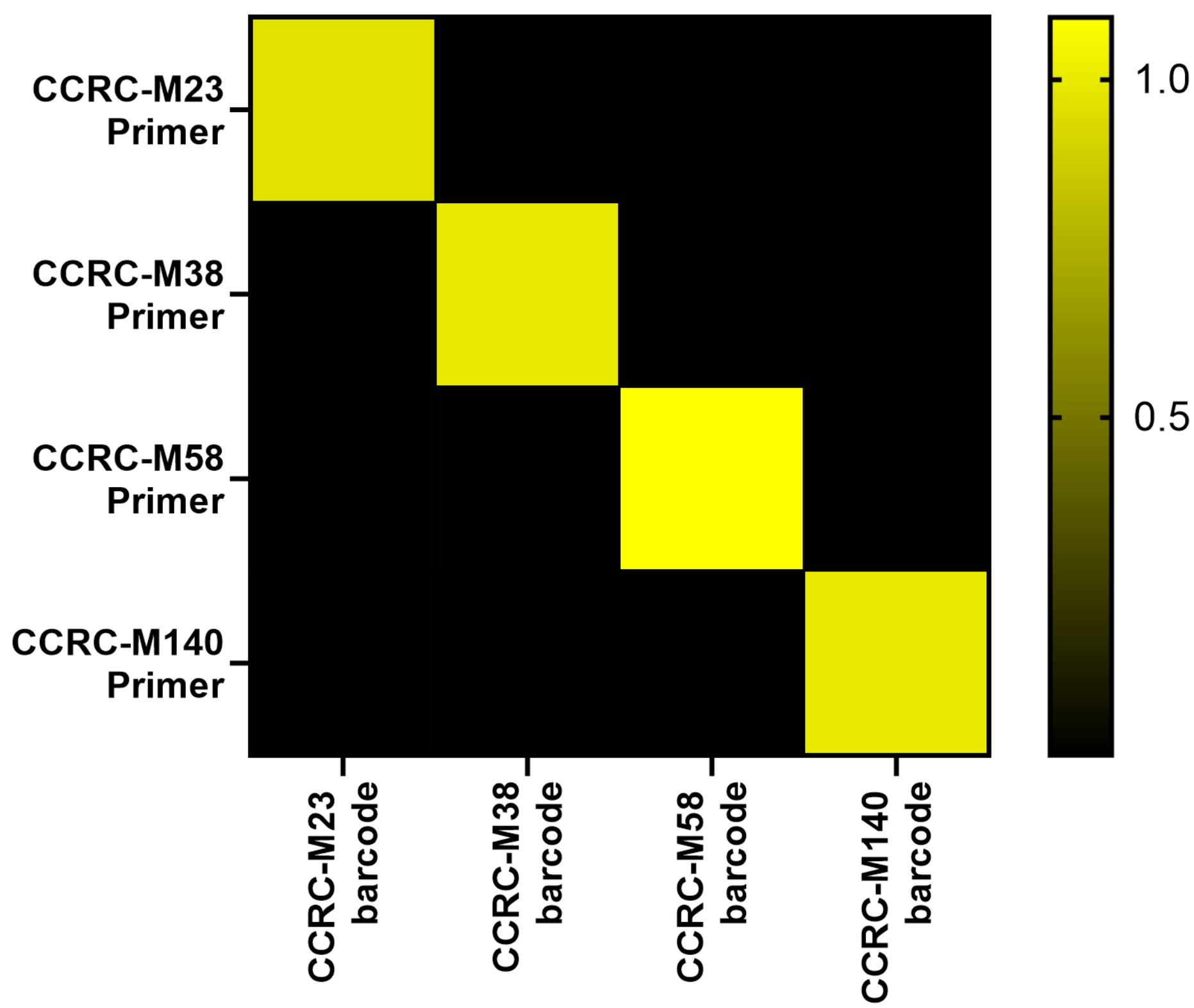

Supplemental Figure S2: Barcode primer set amplification specificity: Primer pairs (500 nM per primer) specific for the barcode sequences of CCRC-M23, CCRC-M38, CCRC-M58, and CCRC-M140 were incubated with 2 pg of isolated DNA barcode sequence under standard qPCR conditions as described in Materials and Methods. A heat map is shown of the linear amplicon abundance values relative to the target barcode sequence resulting from an all-by-all comparison of these amplification trials. Relative abundance values are colored according to the scale shown on the right. Average relative abundance values are shown (n = 3).

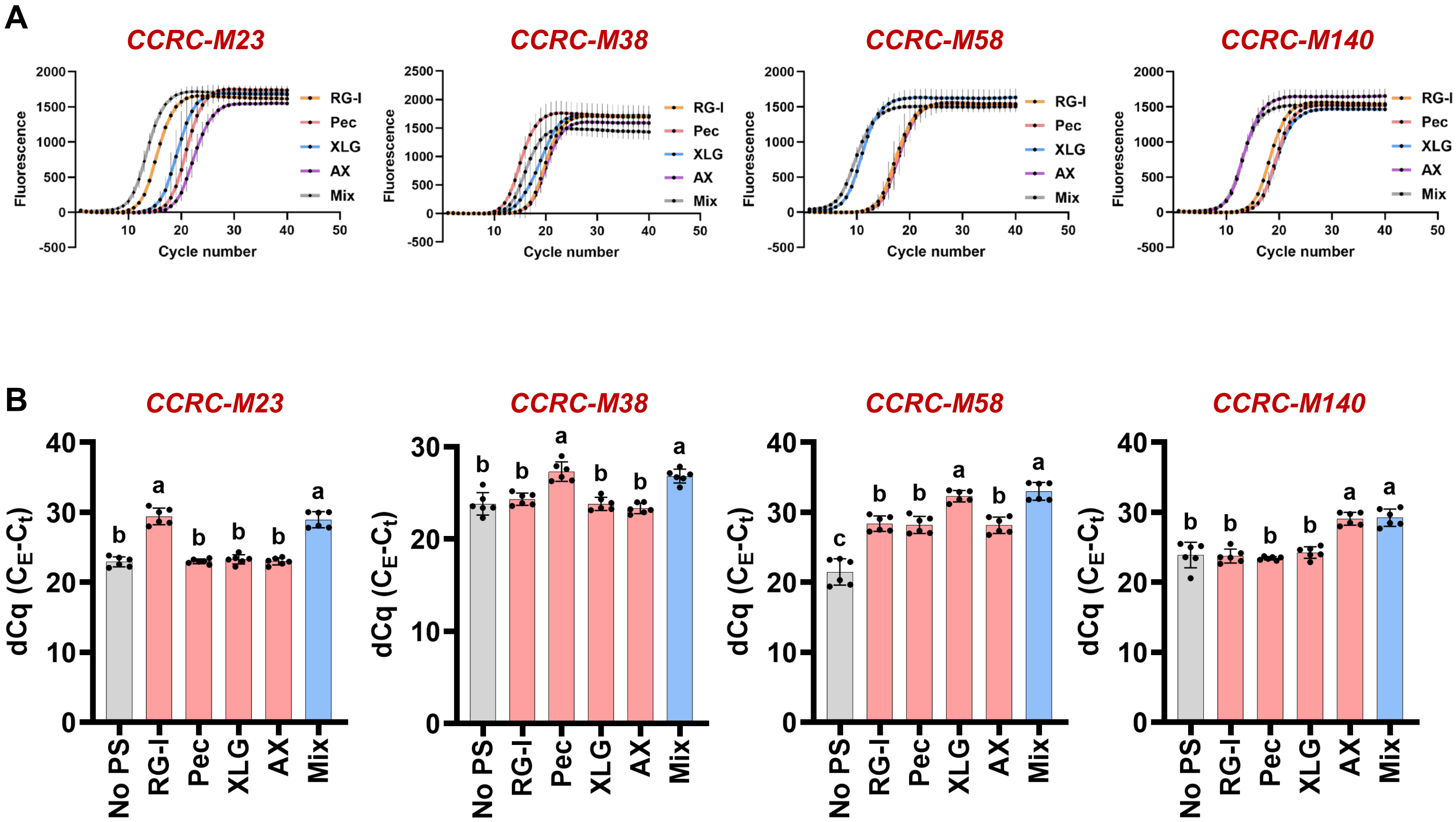

Supplemental Figure S3. Amplification curves and cycle times of antibody selectivity experiment. A. Representative amplification curves are shown for the indicated antibody applied to soy RG-I (RG-I; orange line), apple pectin (Pec; red line), tamarind xyloglucan (XLG; blue line), wheat arabinoxylan (AX; purple line), or an equal part mixture of these polysaccharides (Mix; gray line). Error bars represent SD (n = 3). **B.** The dCq values (Max cycle- Cq value) extracted from amplification curves resulting from each polysaccharide being probed by the indicated antibody are shown. Error bars represent SD (n = 6; all points shown). Letters above bars represent statistically different categories as evaluated by One-Way ANOVA analysis followed by Tukey's post-hoc analysis. Letters indicate sample categories that are statistically different from one another (P<0.0001). Samples sharing the same letter are not statistically different from each another.

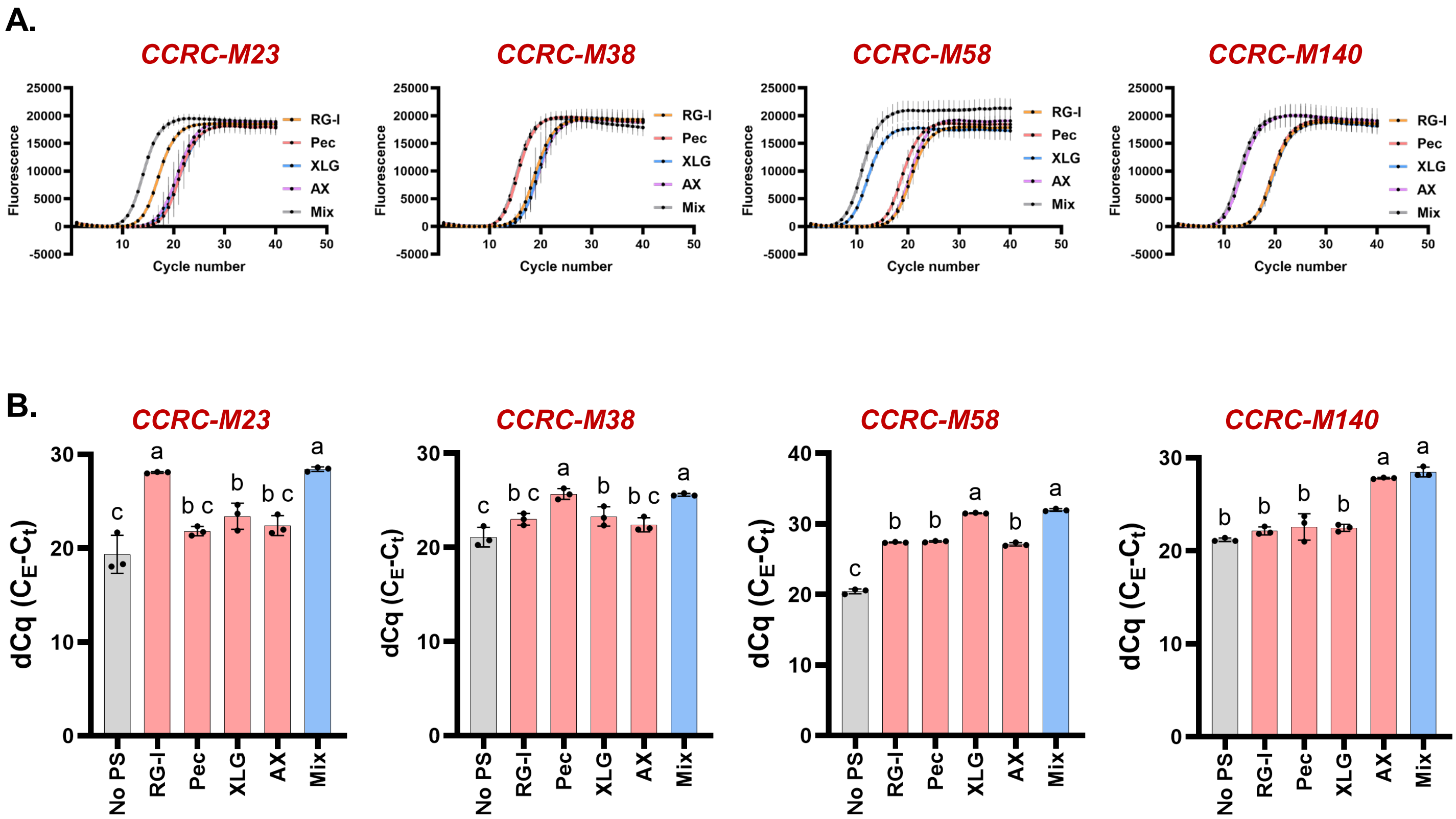

Supplemental Figure S4: Antigen specificity analysis of barcoded antibodies: A. 10 ng of carbohydrate antigens or an equal mass mixture (Mix) were arrayed replicate wells of a 96-well PCR plate and then probed with pooled DNA barcoded antibodies. After removing unbound antibody, barcode-specific primers were added to each well and bound antibody barcodes were quantified by qPCR. **B.** Representative amplification curves are shown for the indicated barcode primer set applied to soy RG-I (RG-I; orange line), apple pectin (Pec; red line), tamarind xyloglucan (XLG; blue line), wheat arabinoxylan (AX; purple line), or an equal part mixture of these polysaccharides (Mix; gray line). Error bars represent SD (n = 3). **C.** The dCq values (Max cycle- Cq value) extracted from amplification curves resulting from each polysaccharide being probed by the indicated antibody are shown. A no polysaccharide (No PS) negative control was included in each experiment. Error bars represent SD (n = 3; all points shown). Letters above bars represent statistically different categories as evaluated by One-Way ANOVA analysis followed by Tukey's post-hoc analysis. Samples labeled with different letters are statistically different from one another ($P<0.001$). Samples sharing the same letter are not statistically different from one another.

Supplemental Table I: DNA barcode sequences used in this study

mAb designation[a]	Barcode sequence (5'-3')[b]
CCRC-M23	GTAAAACGACGGCCAG*CTCATACTGGTTTGAAGTGG*GTCATAGCTGTTTCCTG*CCCATGACAGGGTGCTAAAG*
CCRC-M38	GTAAAACGACGGCCAG*CACTTATTAGGAGGCAGATG*GTCATAGCTGTTTCCTG*GTACCTACGATCCTAGACAT*
CCRC-M58	GTAAAACGACGGCCAG*TATGCGACGCCCGTACTTTG*GTCATAGCTGTTTCCTG*AAACCCGCAGGATGCAGATG*
CCRC-M140	GTAAAACGACGGCCAG*TACTACCGGGCATGGATGAT*GTCATAGCTGTTTCCTG*AATGTCAGTTCACCGGCGCT*

[a]The antibody to which the corresponding barcode was attached is indicated. [b] The sequence of the attached DNA barcode is shown from 5'-3' with the unique sequences underlined and in italics.

Supplemental Table II: Oligonucleotide primers used in this study

Primer name[a]	Primer sequence (5'-3')[b]	Purpose[c]
M13 Forward	GTAAAACGACGGCCAG	General barcode amplification
M13 Reverse	CAGGAAACAGCTATGAC	General barcode amplification
CCRC-M23 BC Forward	CTCATACTGGTTTGAAGTGG	Selective barcode amplification
CCRC-M23 BC Reverse	CTTTAGCACCCTGTCATGGG	Selective barcode amplification
CCRC-M38 BC Forward	CACTTATTAGGAGGCAGATG	Selective barcode amplification
CCRC-M38 BC Reverse	ATGTCTAGGATCGTAGGTAC	Selective barcode amplification
CCRC-M58 BC Forward	TATGCGACGCCCGTACTTTG	Selective barcode amplification
CCRC-M58 BC Reverse	CATCTGCATCCTGCGGGTTT	Selective barcode amplification
CCRC-M140 BC Forward	TACTACCGGGCATGGATGAT	Selective barcode amplification
CCRC-M140 BC Reverse	AGCGCCGGTGAACTGACATT	Selective barcode amplification

[a]Primer names, [b]5'→3' sequences of primers, and [c]their purpose in this study are shown.
